# Mixed Finite Element Formulation for Navier–Stokes Equations for Magnetic Effects on Biomagnetic Fluid in a Rectangular Channel

**DOI:** 10.3390/ma15082865

**Published:** 2022-04-13

**Authors:** Erwan Hafizi Kasiman, Ahmad Beng Hong Kueh, Airil Yasreen Mohd Yassin, Norsarahaida Saidina Amin, Mugahed Amran, Roman Fediuk, Evgenii Vladimirovich Kotov, Gunasekaran Murali

**Affiliations:** 1 School of Civil Engineering, Faculty of Engineering, Universiti Teknologi Malaysia (UTM), Johor Bahru 81310, Malaysia; erwanhafizi@utm.my; 2 Department of Civil Engineering, Faculty of Engineering, Universiti Malaysia Sarawak, Kota Samarahan 94300, Malaysia; 3 UNIMAS Water Centre (UWC), Faculty of Engineering, Universiti Malaysia Sarawak, Kota Samarahan 94300, Malaysia; 4 School of Energy, Geoscience, Infrastructure and Society, Heriot-Watt University Malaysia Campus, Putrajaya 62200, Malaysia; a.mohd_yassin@hw.ac.uk; 5 Department of Mathematical Sciences, Faculty of Science, Universiti Teknologi Malaysia (UTM), Johor Bahru 81310, Malaysia; norsarahaida@utm.my; 6 Department of Civil Engineering, College of Engineering, Prince Sattam Bin Abdulaziz University, Alkharj 16273, Saudi Arabia; 7 Department of Civil Engineering, Faculty of Engineering and IT, Amran University, Amran 9677, Yemen; 8 Polytechnic Institute, Far Eastern Federal University, Vladivostok 690922, Russia; fedyuk.rs@dvfu.ru; 9 Peter the Great St. Petersburg Polytechnic University, St. Petersburg 195251, Russia; ekotov.cfd@gmail.com (E.V.K.); murali_22984@yahoo.com (G.M.)

**Keywords:** mixed formulation, finite element, biomagnetic fluid dynamic, Navier–Stokes, computational simulation

## Abstract

The article presents the mixed finite element formulation for examining the biomagnetic fluid dynamics as governed by the Navier–Stokes equation, coupled with energy and magnetic expressions. Both ferrohydrodynamics and magnetohydrodynamics describe the additional magnetic effects. For model discretization, the Galerkin weighted residual method was performed. Departing from a good agreement with existing findings, a biomagnetic flow (blood) in a straight rectangular conduit was then simulated in the presence of a spatially changing magnetic distribution. By virtue of negligible spatial variation influence from the magnetic field, the effects of Lorentz force were not presently considered. It was further found that the model accurately exhibits the formation and distribution of vortices, temperature, and skin friction located adjacent to and remotely from the source of magnetic load following a rise in the magnetic intensity.

## 1. Introduction

In the past decades, the interaction of fluid in motion with the magnetic field is among the most prominent studies conducted due to its vast applications in engineering, astrophysics, medicine, and many other fields. Particularly in the field of biomedical sciences, the potential for applications of the interaction between biofluid and the magnetic source is wide-ranging. To name a few, cancer-remedying drug carriers, magnetic hyperthermia for wound and cancer treatments, magnetic tracers, cell separation, and magnetic particles targeted drug implementation are some fertile applications of magnetic fluid dynamics [1,2,3,4,5]. Therefore, it is an ever-broadening area that has driven a vast amount of research in recent years, pending a diversity of technological developments, discoveries, and harvestings [6,7]. Two rapidly matured fields, ferrohydrodynamics (FHD) and magnetohydrodynamics (MHD), are research domains that are imperative to the formation of the underlying theory in the computational investigation of biomagnetic fluid dynamics (BFD) in the present study. FHD is devoted to non-electrically conducted magnetic fluid flowing in the presence of a magnetic field [8], whereas MHD is concerned with electrically conducted movement within the magnetic field [9].

BFD, which is a special branch of fluid dynamics governed by both FHD and MHD, has gathered strong research interest in the past few years. Biomagnetic fluid is defined as fluid in the living creature (humans and animals) that can interact with the external occurrence of a magnetic field. Blood is one of the biomagnetic fluids due to its iron oxide content in the form of hemoglobin that reacts with the magnetic field. The field of BFD has emerged with the intent to provide an understanding of the behavior of biomagnetic fluid that is subjected to the magnetic field for mechanical, medicinal, and bioengineering applications.

Blood is generally defined as the biological liquid that navigates the heart, veins, arteries, and capillaries of vertebrates to transport oxygen and nourishment around the circuitry within the body, and to carry waste away. About 8% of the human weight is blood. Magnetic susceptibility measures how intensely a material reacts to the applied magnetic force field. The presence of iron oxide in the red blood cells is what makes the blood react to the magnetic field. The red blood cell exhibits both positive and negative magnetic susceptibilities, such that oxygenated and deoxygenated red blood cells are characterized with values of −1.6 × 10^−7^ and 3.5 × 10^−6^. Oxygenated blood is diamagnetic, or repellant to magnets, whilst the reverse is true for deoxygenated blood, which is paramagnetic. Investigations have been conducted to determine how numerous magnetic field influences on the human body can be attributed to these characteristics. Higashi et al. [10] showed that the red blood cell orients its disk principal plane so that it aligns to the magnetic field, which in turn changes the blood viscosity. Haik et al. [11] interpreted that the changes in the blood viscosity are due to the increase in the apparent viscosity of the blood when subjected to the magnetic field. This theory was supported by experiments showing that the blood flow rate dropped by 30% when a huge intensity of 10 Tesla was imposed by the magnetic field. Ruuge and Ruestri [12] suggested that the red blood cells could be used as drug carriers by chemically binding the drug to the red blood cells, which can then be directed to a particular location in the body by a magnet source.

In terms of BFD, Haik [13] pioneered the development of several works that mathematically modeled the biomagnetic fluid (blood) in the existence of the magnetic field. Considering the anisotropic property of blood when exposed to the magnetic field, the Stokes’ principle, which was used to derive the Navier–Stokes equation, was modified by the author to take the effects of the magnetic field into consideration. It is worth noting that the mathematical expression of BFD formulated by Haik [13] only considered the magnetization accredited to the magnetic field, but ignored the Lorentz force because the blood was assumed to be a non-electrically conducting fluid. The work was then extended by Haik et al. [14] to examine the effects of the magnetic intensity on the biomagnetic flow in a channel with a thrombus by using the finite analytic method. They concluded that when the biomagnetic liquid is exposed to an external magnetic field, its behavior varies considerably in terms of occurrence of recirculation regions and in an increase in the friction coefficient. Loukopoulos and Tzirtzilakis [15] studied the biomagnetic channel flow in spatially varying magnetic fields by considering the FHD principle while treating the biomagnetic fluid as a non-conducting medium. The same model was adopted in a study by Tzirtzilakis and Loukopoulos [16], where an additional effect due to Lorentz force was considered for both uniform and localized magnetic fields. Apart from difficulties in imposing the vorticity boundary conditions, their employed finite difference method yielded a satisfactory convergence. Both works [15,16] observed the formation of a vortex in the fluid and noted rises in vortex size, temperature, heat transfer rate, and skin friction at the wall at the magnetic source location, with varying intensities. Mousavi et al. [17] explored BFD for a Newtonian flow within a confined vessel due to the effects of a non-uniformly distributed magnetic field. Shit and Roy [18] modeled the blood flow through the nonlinear micropolar fluid model under a very low Reynolds number and noted that the assigned magnetic load intensified the flow’s microrotation component, thereby enhancing the resulting pressure gradient. Siddiqa et al. [19] examined the magnetic field- and thermal radiation-inflicted impacts on the fluid behaviors of a conducting Newtonian flow inside a rectangular duct. Strek and Jopek [20] simulated the time-affected heat transfer of ferrofluid in a conduit fluid due to the magnetic dipole by adopting the FHD principle, without the inclusion of Lorentz force. They noticed the formation of a vortex near the magnetic field initiation point, with the cooler ferrofluid displacing the hotter. It was once again noticed that the maximum flow velocity rose with the magnetic strength. Simulated with the thermal and velocity slip conditions, Soomro et al. [21] inspected the MHD of Williamson nanofluid flows on a vertically translated face. BFD for electrically conducting blood in a symmetrical stenosed blood channel was modeled by Murtaza et al. [22]. Tzirtzilakis et al. [23] investigated the effects of the magnetic field in the biomagnetic fluid flow in a 3D rectangular duct with the FHD principle. The effects of temperature were neglected while the biomagnetic fluid was considered to be non-conducting. It was observed that increments in the magnetic field strength reduced the flow rate by up to 40% for a strong magnetic field. For a spatially varying magnetic field, secondary flow occurred in the transverse plane in the form of two rotating vortices. Furthermore, they noticed that there was no secondary flow for a uniform magnetic field, but the axial velocity reduced to about 10% in the strong magnetic field. The influence of a magnetic field on the flow in a horizontally bent duct was numerically studied by Akar et al. [24], employing the ANSYS computer solver. With FHD, Wang et al. [25] conducted a numerical simulation of blood flow under the influence of a magnetic field to study magnetic targeting drug delivery. They reported that, after imposing the magnetic field, the velocity of the flow decreased, whilst the pressure drop increased at the target location. In the study of the biomagnetic fluid flow in a channel with stenosis subjected to magnetic field, the temperature on the upper plate downstream the stenosis increased, whilst overall temperature downstream the stenosis was kept cooler [26]. The velocity and temperature fields were affected considerably for a moderate stenosis of 60%, even for a very low magnetic intensity. 

Kenjeres and Opdam [27] conducted a numerical simulation of realistic arteries subjected to strong non-uniform magnetic fields, with the argument that the Tzirtzilakis and Loukopoulos [16] model lacked one term: electrical potential. They solved the governing equations by applying a finite volume second-order Navier–Stokes/Maxwell solver in structured multi-block non-orthogonal geometries, including the effects of magnetization, Lorentz force, and electrical potential, forming the conclusion that with the magnetic field, significant pressure changes occur at locations with high magnetic field strength. The secondary flow patterns were also noticed at the location of the magnetic field source, as replicated in [28]. The blood was considered a non-Newtonian fluid by Li and Hung [27] with a viscosity term described by the power law model, demonstrating that the same shear stress pattern on the wall compared to the Newtonian model was observed. The introduction of a magnetic field breaks the symmetry downstream of the flow and enlarges the vortex close to the magnetic field source, as observed by Tzirtzilakis [26]. Habibi and Ghasemi [29], treating the blood as non-conducting but containing magnetization properties, investigated the effects of a high-gradient magnetic field on the concentration of magnetic nanoparticles in the blood, adopting the finite volume method with the SIMPLE scheme as the solver. Their results showed that when the magnetic field is applied to the blood, the concentration of the nanoparticles increases and the particles create an obstacle in front of the blood flow, resulting in an increase in the blood velocity. Singh et al. [30] simulated a viscoelastic, steady MHD flow with the convective term over a vertical surface, under magnetized and convective heat, and reported that the inherent viscoelasticity had enhancing effects on the magnetized vertical surface current density, but reacted conversely with the magnetic diffusivity.

From the works reviewed above, it can be recognized that the numerical studies of BFD mainly involved either the employment of finite difference or finite volume, with little work, thus far, relating to the finite element method. The advantages of the finite element method lie in its generality in treating an arbitrary problem domain [31,32,33,34,35,36]. In considering more realistic fluid behavior, the inclusion of various effects in the current context (the magnetic effects) usually results in coupled partial differential equations. One of the major difficulties in solving such a coupled problem is handling the pressure terms in which singularity occurs. Moreover, the stability of the time integration also poses some problems in obtaining a converged solution. Among available techniques, the mixed finite element formulation is the most appropriate to remedy the aforementioned matters [37]. 

The mixed formulation stems from the problem encountered early in the implementation of FEM for the incompressible Navier–Stokes equation. Employing the Galerkin weighted residual method, the resulting Navier–Stokes equation is an incomplete parabolic equation and has singular behavior. This singular behavior is due to the lack of diffusive terms in the continuity equation. Pressure is considered the unknown but does not relate to any constitutive equation. The continuity equation is the kinematic constraint to the system [38] whilst the pressure acts as the Lagrange multiplier. A non-singular matrix with a zero diagonal must be ensured to circumvent the problem with a global singularity of the whole system. To do so, the selection of interpolation function for velocity and pressure must obey the Ladyzhenskaya–Babuška–Brezzi (LBB) condition. The stabilized mixed formulation can be achieved by selecting interpolation functions for velocity and pressure [39]. Thus, the selection of the Taylor–Hood element, quadratic interpolation for velocity, and linear interpolation for pressure ensures that the formulation is stable whilst keeping the degrees of freedom for each element at a minimum. Furthermore, the element warrants an optimal quadratic convergence, significantly reducing the computational time. Whilst this technique has been employed in many areas, such as in heat transfer analysis and fluid–structure interaction, there has yet to be any attempt to solve matters of biomagnetic fluid flow using such a technique. Therefore, the current article aims to formulate the Navier–Stokes equations, coupled with energy and magnetic fields, using the mixed finite element method. After the introductory section, the relevant formulation for biomagnetic flow is presented. The effects of the magnetic field intensities on the biomagnetic fluid in a rectangular channel are then examined, departing from the verification with existing findings.

## 2. Mixed Formulation Strategy

The mixed finite element formulation for the biomagnetic flow under the influence of spatially varying magnetic fields is first described. In essence, its mathematical governing premise contains the Navier–Stokes equation with heat transfer, plus an additional term that characterizes the magnetic force effects. Additional terms in the Navier–Stokes equation describe the interaction of a magnetized biomagnetic fluid with a spatially alternating magnetic field [40]. It is assumed that the induction in the flow is small so that only the magnetic field affects the flow, but not the other way around. The flow is assumed to be two-dimensional, laminar, incompressible, Newtonian, and non-isothermal. The magnetic force depends on both the magnetic field strength and gradient.

### 2.1. Dimensionless Form of BFD Governing Equations

Introducing the dimensionless variables,

(1) $$x=\frac{\bar{x}}{\bar{h}}\;,\;y=\frac{\bar{y}}{\bar{h}},\;\;\;\;u=\frac{\bar{u}}{\bar{u_{r}}},\;\;\;\;v=\frac{\bar{v}}{\bar{u_{r}}},$$

$$p=\frac{\bar{p}}{\bar{\rho }{\bar{u_{r}}}^{2}}\;,\;H=\frac{\bar{H}}{\bar{H_{r}}},\;\;\;\;T=\frac{\bar{T_{u}}-\bar{T}}{\bar{\Delta T}},$$

where the BFD governing equations is cast into dimensionless governing equations, in which variables with the overhead bar are those in their dimensional forms. Here, $\bar{h}$ is the reference length; $\bar{u_{r}}$ is the reference velocity; $\bar{H_{r}}$ is reference magnetic field intensity; $\bar{\Delta T}=\bar{T_{u}}-\bar{T_{l}}$ is the temperature difference; $\bar{T_{u}}$ is the temperature of the upper plate; $\bar{T_{l}}$ is the temperature of the lower plate; $\bar{x}$ and $\bar{y}$ are the dimensional lengths; $\bar{u}$ and $\bar{v}$ are the dimensional velocities; $\bar{H}$ is the dimensional magnetic field gradient; $\bar{\rho }$ is the density; and $\bar{p}$ is the dimensional pressure. The governing equations for the BFD model in the dimensionless form are, hence, for Ω ⊂ ℛ^2^:

(2) $$\frac{\partial u}{\partial x}+\frac{\partial v}{\partial y}=0\;in\;\Omega \;$$

(3) $$\left(u\frac{\partial u}{\partial x}+v\frac{\partial u}{\partial y}\right)+\frac{\partial p}{\partial x}-Mn_{F}H\frac{\partial H}{\partial x}T-\frac{1}{Re}\left(\frac{\partial^{2}u}{\partial x^{2}}+\frac{\partial^{2}u}{\partial y^{2}}\right)+\frac{Mn_{M}}{Re}H^{2}u=0\;in\;\Omega$$

(4) $$\left(u\frac{\partial v}{\partial x}+v\frac{\partial v}{\partial y}\right)+\frac{\partial p}{\partial y}-Mn_{F}H\frac{\partial H}{\partial y}T-\frac{1}{Re}\left(\frac{\partial^{2}v}{\partial x^{2}}+\frac{\partial^{2}v}{\partial y^{2}}\right)=0\;in\;\Omega$$

(5) $$-\left(u\frac{\partial T}{\partial x}+v\frac{\partial T}{\partial y}\right)-Mn_{F}E_{c}\left(uH\frac{\partial H}{\partial x}+vH\frac{\partial H}{\partial y}\right)\left(\varepsilon -T\right)-E_{c}\frac{Mn_{M}}{Re}H^{2}u^{2}\;+\frac{1}{Re\;P_{r}}\left(\frac{\partial^{2}T}{\partial x^{2}}+\frac{\partial^{2}T}{\partial y^{2}}\right)-\frac{E_{c}}{Re}\left[2{\left(\frac{\partial u}{\partial x}\right)}^{2}+2{\left(\frac{\partial v}{\partial y}\right)}^{2}+{\left(\frac{\partial v}{\partial x}+\frac{\partial u}{\partial y}\right)}^{2}\right]=0\;in\;\Omega$$

Furthermore, the extra dimensionless expressions are:

(6) $$\begin{array}{cc} \mathrm{Reynolds}\mathrm{number}: & R_{e}=\frac{\bar{\rho }{\bar{u}}_{r}\bar{h}}{\bar{\mu }}\\ \mathrm{Eckert}\mathrm{number}: & E_{c}=\frac{{{\bar{u}}_{r}}^{2}}{\bar{c_{p}}\bar{\Delta T}}\\ \mathrm{Prandtl}\mathrm{number}: & P_{r}=\frac{\bar{\mu c_{p}}}{\bar{k}}\\ \mathrm{Temperature}\mathrm{number}: & \varepsilon =\bar{\frac{T_{u}}{\bar{\Delta T}}}\\ \mathrm{Magnetic}\mathrm{number}\mathrm{due}\mathrm{to}\mathrm{FHD}: & Mn_{F}=\frac{\bar{\mu_{o}}\bar{K}{\bar{H_{r}}}^{2}\bar{\Delta T}}{\rho {\bar{u_{r}}}^{2}}\\ \mathrm{Magnetic}\mathrm{number}\mathrm{due}\mathrm{to}\mathrm{MHD}: & Mn_{M}=\frac{\bar{\sigma }{\bar{\mu_{o}}}^{2}{\bar{H_{r}}}^{2}{\bar{h}}^{2}}{\mu } \end{array}$$

where $\bar{\mu }$ is the viscosity of the fluid; $\;\bar{\sigma }$ is the electrical conductivity; $\bar{c_{p}}$ is the specific heat; and *k* is the thermal conductivity.

### 2.2. Discretization of BFD Governing Equations

The Galerkin method seeks to solve the unknowns of a function by forming a weighted average of the error and forcing this weighted average to vanish. To discretize Equations (2)–(5) by the Galerkin method, the variables are first approximated for Ω ⊂ ℛ^2^ as:

(7) $$u\left(x,y\right)=\overset{m}{\underset{j=1}{\sum }}N_{j}\left(x,y\right)u_{j}\;v\left(x,y\right)=\overset{m}{\underset{j=1}{\sum }}N_{j}\left(x,y\right)v_{j}\;p\left(x,y\right)=\overset{n}{\underset{j=1}{\sum }}L_{j}\left(x,y\right)p_{j}\;T\left(x,y\right)=\overset{m}{\underset{j=1}{\sum }}N_{j}\left(x,y\right)T_{j}$$

where $N_{j}\left(x,y\right)$ and $L_{j}\left(x,y\right)$ denote the quadratic and linear Lagrange shape functions, respectively. These shape functions correspond to different finite element meshes over the same domain, Ω. The term mixed formulation comes from the fact that different shape function orders are used for the variables’ approximations. A weighting function, w(x,y), for both the momentum and energy equations is given as:

(8) $$w_{i}\left(x,y\right)=N_{i}\left(x,y\right)$$

whilst for the continuity equation,

(9) $$w_{i}\left(x,y\right)=L_{i}\left(x,y\right)$$

where the index i is the number of the equation that equals the total degree of freedom of the respective shape function.

Employing the Galerkin method to Equations (2)–(5):

(10) $$\int_{\Omega }L_{i}\frac{\partial u}{\partial x}+L_{i}\frac{\partial v}{\partial y}d\Omega =0$$

(11) $$\int_{\Omega }N_{i}\left(u\frac{\partial u}{\partial x}+v\frac{\partial u}{\partial y}\right)+N_{i}\frac{\partial p}{\partial x}-Mn_{F}H_{M}\frac{\partial H}{\partial x}N_{i}T-\frac{1}{Re}N_{i}\left(\frac{\partial^{2}u}{\partial x^{2}}+\frac{\partial^{2}u}{\partial y^{2}}\right)\;+\frac{Mn_{M}}{Re}H^{2}N_{i}ud\Omega =0$$

(12) $$\int_{\Omega }N_{i}\left(u\frac{\partial v}{\partial x}+v\frac{\partial v}{\partial y}\right)+N_{i}\frac{\partial p}{\partial y}-Mn_{F}H\frac{\partial H}{\partial y}N_{i}T-\frac{1}{Re}N_{i}\left(\frac{\partial^{2}v}{\partial x^{2}}+\frac{\partial^{2}v}{\partial y^{2}}\right)d\Omega =0$$

(13) $$\int_{\Omega }-N_{i}\left(u\frac{\partial T}{\partial x}+v\frac{\partial T}{\partial y}\right)-Mn_{F}E_{c}N_{i}\left(uH\frac{\partial H}{\partial x}+vH\frac{\partial H}{\partial y}\right)\left(\varepsilon -T\right)\;-E_{c}\frac{Mn_{M}}{Re}H^{2}N_{i}u^{2}+\frac{1}{Re\;P_{r}}N_{i}\left(\frac{\partial^{2}T}{\partial x^{2}}+\frac{\partial^{2}T}{\partial y^{2}}\right)\;-\frac{E_{c}}{Re}N_{i}\left[2{\left(\frac{\partial u}{\partial x}\right)}^{2}+2{\left(\frac{\partial v}{\partial y}\right)}^{2}+{\left(\frac{\partial v}{\partial x}+\frac{\partial u}{\partial y}\right)}^{2}\right]d\Omega =0$$

Exploiting integration by parts and applying it to Equations (10)–(13) produces

(14) $$\int_{\Omega }L_{i}\frac{\partial u}{\partial x}+L_{i}\frac{\partial v}{\partial y}d\Omega =0$$

(15) $$\int_{\Omega }N_{i}\left(u\frac{\partial u}{\partial x}+v\frac{\partial u}{\partial y}\right)+N_{i}\frac{\partial p}{\partial x}-Mn_{F}H\frac{\partial H}{\partial x}N_{i}T+\frac{1}{Re}\frac{\partial N_{i}}{\partial x}\left(\frac{\partial u}{\partial x}+\frac{\partial u}{\partial y}\right)\;+\frac{Mn_{M}}{Re}H^{2}N_{i}ud\Omega =\oint_{s}N_{i}\left[\frac{1}{Re}\left(\frac{\partial u}{\partial x}+\frac{\partial u}{\partial y}\right)\right]ds$$

(16) $$\int_{\Omega }N_{i}\left(u\frac{\partial v}{\partial x}+v\frac{\partial v}{\partial y}\right)+N_{i}\frac{\partial p}{\partial y}-Mn_{F}H\frac{\partial H}{\partial y}N_{i}T-\frac{1}{Re}\frac{\partial N_{i}}{\partial y}\left(\frac{\partial v}{\partial x}+\frac{\partial v}{\partial y}\right)d\Omega \;=\oint_{s}N_{i}\left[\frac{1}{Re}\left(\frac{\partial v}{\partial x}+\frac{\partial v}{\partial y}\right)\right]ds$$

(17) $$\begin{array}{ll} \int_{\Omega }-N_{i}(u\frac{\partial T}{\partial x}+ & v\frac{\partial T}{\partial y})-Mn_{F}E_{c}N_{i}\left(uH\frac{\partial H}{\partial x}+vH\frac{\partial H}{\partial y}\right)\left(\varepsilon -T\right)\\ & -\frac{1}{Re\;P_{r}}\left(\frac{\partial N_{i}}{\partial x}\frac{\partial T}{\partial x}+\frac{\partial N_{i}}{\partial y}\frac{\partial T}{\partial y}\right)\\ & -\frac{E_{c}}{Re}N_{i}\left[2{\left(\frac{\partial u}{\partial x}\right)}^{2}+2{\left(\frac{\partial v}{\partial y}\right)}^{2}+{\left(\frac{\partial v}{\partial x}+\frac{\partial u}{\partial y}\right)}^{2}\right]d\Omega \\ & =\oint_{s}N_{i}\left[\frac{1}{Re}\left(\frac{\partial T}{\partial x}+\frac{\partial T}{\partial y}\right)\right]ds\\ & +\int_{\Omega }E_{c}\frac{Mn_{M}}{Re}H^{2}N_{i}u^{2}\\ & \;+\frac{E_{c}}{Re}N_{i}\left[2{\left(\frac{\partial u}{\partial x}\right)}^{2}+2{\left(\frac{\partial v}{\partial y}\right)}^{2}+{\left(\frac{\partial v}{\partial x}+\frac{\partial u}{\partial y}\right)}^{2}\right]d\Omega \end{array}$$

Substituting Equation (7) into Equations (14)–(17) results in the final forms of the finite element system:

(18) $$\int_{\Omega }L_{i}\frac{\partial N_{j}}{\partial x}u_{j}+L_{i}\frac{\partial N_{j}}{\partial y}u_{j}d\Omega =0$$

(19) $$\int_{\Omega }N_{i}\left(u\frac{\partial N_{j}}{\partial x}u_{j}+v\frac{\partial N_{j}}{\partial y}u_{j}\right)+N_{i}\frac{\partial L_{j}}{\partial x}p_{j}-Mn_{F}H\frac{\partial H}{\partial x}N_{i}N_{j}T_{j}+\frac{1}{Re}\left(\frac{\partial N_{i}}{\partial x}\frac{\partial N_{j}}{\partial x}u_{j}+\frac{\partial N_{i}}{\partial x}\frac{\partial N_{j}}{\partial y}u_{j}\right)\;+\frac{Mn_{M}}{Re}H^{2}N_{i}N_{j}u_{j}d\Omega =\oint_{s}N_{i}\left[\frac{1}{Re}\left(\frac{\partial u}{\partial x}+\frac{\partial u}{\partial y}\right)\right]ds$$

(20) $$\int_{\Omega }N_{i}\left(u\frac{\partial N_{j}}{\partial x}v_{j}+v\frac{\partial N_{j}}{\partial y}v_{j}\right)+N_{i}\frac{\partial L_{j}}{\partial y}p_{j}-Mn_{F}H\frac{\partial H}{\partial y}N_{i}N_{j}T_{j}-\frac{1}{Re}\left(\frac{\partial N_{i}}{\partial y}\frac{\partial N_{j}}{\partial x}v_{j}+\frac{\partial N_{i}}{\partial y}\frac{\partial N_{j}}{\partial y}v_{j}\right)d\Omega \;=\oint_{s}N_{i}\left[\frac{1}{Re}\left(\frac{\partial v}{\partial x}+\frac{\partial v}{\partial y}\right)\right]ds$$

(21) $$\int_{\Omega }-N_{i}\left(u\frac{\partial N_{j}}{\partial x}T_{j}+v\frac{\partial N_{j}}{\partial y}T_{j}\right)-Mn_{F}E_{c}N_{i}\left(uH\frac{\partial H}{\partial x}+vH\frac{\partial H}{\partial y}\right)\left(\varepsilon -N_{j}T_{j}\right)-\frac{1}{Re\;P_{r}}\left(\frac{\partial N_{i}}{\partial x}\frac{\partial N_{j}}{\partial x}T_{j}+\frac{\partial N_{i}}{\partial y}\frac{\partial N_{j}}{\partial y}T_{j}\right)d\Omega \;=\oint_{s}N_{i}\left[\frac{1}{Re}\left(\frac{\partial T}{\partial x}+\frac{\partial T}{\partial y}\right)\right]ds\int_{\Omega }E_{c}\frac{Mn_{M}}{Re}H^{2}N_{i}u^{2}\;+\frac{E_{c}}{Re}N_{i}\left[2{\left(\frac{\partial u}{\partial x}\right)}^{2}+2{\left(\frac{\partial v}{\partial y}\right)}^{2}+{\left(\frac{\partial v}{\partial x}+\frac{\partial u}{\partial y}\right)}^{2}\right]d\Omega$$

Writing Equations (18)–(21) in a more compact matrix form yields:

(22) $$\left[\begin{array}{cccc} k_{11} & 0 & k_{13} & k_{14}\\ 0 & k_{22} & k_{23} & k_{24}\\ k_{31} & k_{32} & 0 & 0\\ 0 & 0 & 0 & k_{44} \end{array}\right]\left\{\begin{array}{c} u\\ v\\ p\\ T \end{array}\right\}=\left\{\begin{array}{c} 0\\ b_{t}\\ b_{t}\\ f_{4}+b_{t} \end{array}\right\}$$

Note that $b_{t}$ are the boundary terms that arise from the integration by parts procedure. Terms in Equation (22) can be further expressed as:

(23) $$k_{11}=C_{x}+C_{y}+\frac{1}{Re}\left(K_{x}+K_{y}\right)+\frac{Mn_{M}}{Re}S_{c}\;k_{13}=Q_{x}\;k_{14}=-Mn_{F}S_{x}\;k_{22}=C_{x}+C_{y}+\frac{1}{Re}\left(K_{x}+K_{y}\right)\;k_{23}=Q_{y}\;k_{24}=-Mn_{F}S_{y}\;k_{31}=Q_{px}\;k_{32}=Q_{py}\;k_{44}=-\left(C_{x}+C_{y}\right)-\frac{1}{Re\;P_{r}}\left(K_{x}+K_{y}\right)+Mn_{F}E_{c}S_{h}\;f_{4}=\frac{E_{c}}{Re}G+Mn_{F}E_{c}\varepsilon S_{t}+E_{c}\frac{Mn_{M}}{Re}S_{u}$$

where

(24) $$C_{x}=\int_{\Omega }N_{i}u\frac{\partial N_{j}}{\partial x}d\Omega \;C_{y}=\int_{\Omega }N_{i}u\frac{\partial N_{j}}{\partial y}d\Omega \;K_{x}=\int_{\Omega }\frac{\partial N_{i}}{\partial x}\frac{\partial N_{j}}{\partial x}d\Omega \;K_{y}=\int_{\Omega }\frac{\partial N_{i}}{\partial y}\frac{\partial N_{j}}{\partial y}d\Omega \;Q_{x}=\int_{\Omega }N_{i}\frac{\partial L_{j}}{\partial x}d\Omega \;Q_{y}=\int_{\Omega }N_{i}\frac{\partial L_{j}}{\partial y}d\Omega \;Q_{px}=\int_{\Omega }L_{i}\frac{\partial N_{j}}{\partial x}d\Omega \;Q_{py}=\int_{\Omega }L_{i}\frac{\partial N_{j}}{\partial y}d\Omega \;S_{x}=\int_{\Omega }N_{i}\left(H\frac{\partial H}{\partial x}\right)N_{j}d\Omega \;S_{y}=\int_{\Omega }N_{i}\left(H\frac{\partial H}{\partial y}\right)N_{j}d\Omega \;S_{h}=\int_{\Omega }N_{i}\left(uH\frac{\partial H}{\partial x}+vH\frac{\partial H}{\partial y}\right)N_{j}d\Omega \;S_{c}=\int_{\Omega }N_{i}H^{2}N_{j}d\Omega \;G=\int_{\Omega }N_{i}\left(2{\left(\frac{\partial u}{\partial x}\right)}^{2}+2{\left(\frac{\partial v}{\partial y}\right)}^{2}+{\left(\frac{\partial v}{\partial x}+\frac{\partial u}{\partial y}\right)}^{2}\right)d\Omega \;S_{t}=\int_{\Omega }N_{i}\left(uH\frac{\partial H}{\partial x}+vH\frac{\partial H}{\partial y}\right)d\Omega \;S_{u}=\int_{\Omega }N_{i}\left(H^{2}u^{2}\right)d\Omega$$

The Gauss elimination solves the resulting simultaneous equations with pivoting in MATLAB. Note that when the magnetic numbers for FHD and MHD, i.e., $Mn_{F}$ and $Mn_{M}$ are equal to zero, the basic Navier–Stokes equations with heat transfer are recovered.

### 2.3. Biomagnetic Fluid Flow in Rectangular Channels

For demonstrative purposes, the effects of a magnetic field applied to a two-dimensional biomagnetic fluid flow in a straight channel are examined next. The fluid is assumed to be a Newtonian, fully developed, steady, laminar, and incompressible flow. The energy equation is coupled with momentum and continuity equations, thus making the temperature one of the field variables. The properties of blood are adopted to represent the biomagnetic fluid, which flows in a straight rectangular channel.

**Figure 1:**
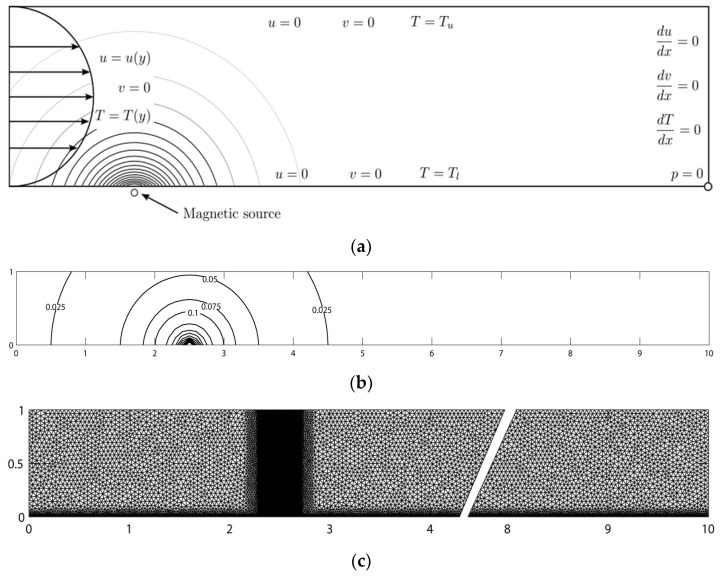
Mesh and boundary condition of biomagnetic flow in channel subjected to a magnetic field. (**a**) Problem domain and boundary condition; (**b**) magnetic field intensity; (**c**) mesh for biomagnetic flow.

Figure 1a illustrates the computational domain and boundary conditions, along with the magnetic source located below the bottom wall of the channel, creating a spatially varying magnetic field. The entrance velocity is assumed as fully developed with the no-slip condition on the top and bottom walls. At the exit, the Neumann boundary condition for the velocity is set as zero. In the mixed finite element formulation, at least one pressure boundary condition is required to ensure a well-defined boundary condition. In the current study, the pressure is imposed at the bottom right corner of the channel. Additionally, the temperature is assigned as having constant values of *T*_u_ and *T*_l_ at the top and bottom walls, respectively. Linear interpolation temperature boundary condition is used at the inlet whilst a similar boundary condition to that of velocity is set at the exit.

## 3. Results and Discussion

### 3.1. Verification

The results from Loukopoulos and Tzirtzilakis [15] for a biomagnetic flow (blood) in the presence of an external magnetic field are considered to verify the developed FE formulation. They considered the blood to have magnetization properties, but behaves as a non-conducting fluid, a condition analogous to the principle of FHD. Additionally, the magnetization was assumed as a linear function of magnetic field strength, *H*, and the temperature difference was $T_{c}-T$, where $T_{c}$ is the Curie temperature. The employed spatially varying magnetic field is such that the magnetic field intensity, *H*, is written in a dimensionless form:

(25) $$H=\frac{-b}{\sqrt{{\left(x-a\right)}^{2}+{\left(y-b\right)}^{2}}}$$

where *b* is the distance from the magnetic source to the bottom of the channel and *a* is the distance from the left wall to the magnetic source. Figure 1b shows the magnetic field contour and the source is located at the point (2.5,0). The magnetic field intensity has the highest value at this point, and its strength weakens as the distance increases. Furthermore, biomagnetic fluid (blood) with a density of 1050 kg/m^3^ and a viscosity of 3.2 × 10^−3^ kg/ms, flowing with a maximum speed of u_r_ = 3.81 × 10^−2^ m/s, is considered. The channel height is *h* = 2.0 × 10^−2^ m with a length ten times the height. From the values provided, the Reynold number (*Re*) is 250.

Apart from *Re*, one important dimensionless parameter is the magnetic number, *Mn_F_*, written as:

(26) $$Mn_{F}=\frac{\bar{\mu_{o}}\bar{K}{\bar{H_{r}}}^{2}\bar{\Delta T}}{\rho {\bar{u_{r}}}^{2}}$$

where $\bar{K}$ is a constant and $\bar{H_{r}}$ is the magnetic field strength at point (2.5,0). In terms of *Re*, it can be rewritten as:

(27) $$Mn_{F}=\frac{\bar{B_{o}}{\bar{M}}_{r}\bar{h^{2}}\bar{\rho }}{\bar{\mu^{2}}\bar{Re^{2}}}$$

where $\bar{B_{o}}$ and ${\bar{M}}_{r}$ are the magnetic induction and the magnetization, respectively, at the point (2.5,0). Loukopoulos and Tzirtzilakis [15] assumed that for the magnetic field of 8T, the blood had reached the full magnetic saturation with the magnetization of 60 A/m. By substituting all the parameters into the magnetic number, the magnetic number is 315 for a magnetic strength of 8 T. Moreover, the temperature difference of $\bar{\Delta T}$ = 39.5 °C was considered with the upper plate value of 43 °C and lower plate value of *T*_l_ = 3.5 °C. For these values of plate temperature, the temperature number ε is 8. Table 1 summarizes other dimensional and dimensionless parameters.

Due to the high-gradient magnetic field used in this study, the Lorentz force does not significantly affect the flow field [40]. Thus, the effect of the Lorentz force governed by the MHD principle is not considered in the remainder of this study. As illustrated in Figure 1c, the computational mesh density is high between *x* = 2 and *x* = 3, since the most significant effect of the magnetic field is found here. The total numbers of elements and nodes are 31,606 and 65,629. With the mixed finite element formulation, each element has 12, 6, and 3 degrees of freedom for velocity, temperature, and pressure, respectively. The sparse matrix technique is employed along with the direct Gauss elimination method for computational efficiency and divergence prevention to circumvent the storage problem for the large matrix.

**Figure 2:**
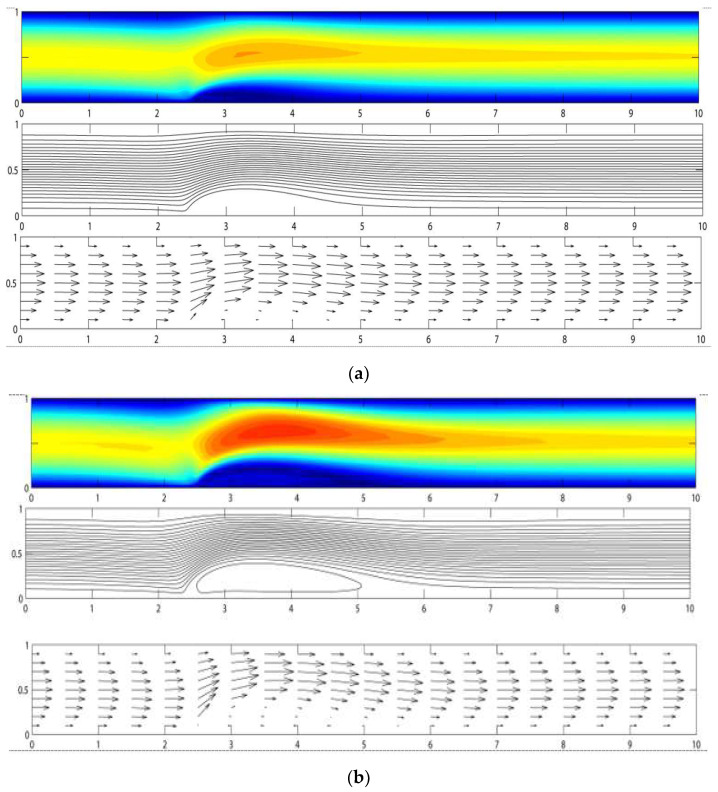

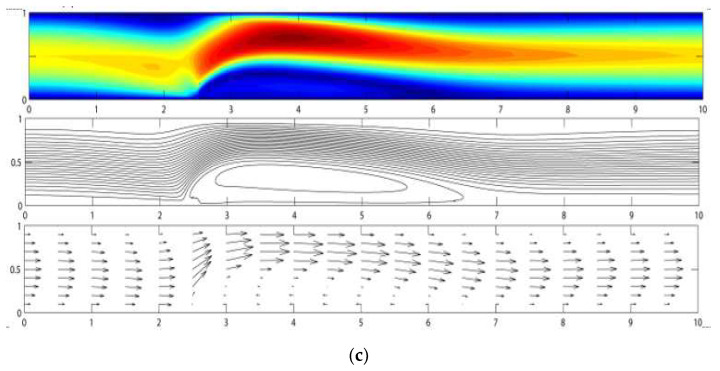
Velocity contour, stream function, and velocity direction for biomagnetic flow subjected to magnetic field strength, $\bar{B_{o}}$, of (**a**) 2 T; (**b**) 4 T; and (**c**) 8 T.

Figure 2a–c show the velocity and stream function contours for the magnetic field strength, $\bar{B_{o}}$, of 2T, 4T, and 8T. The corresponding magnetic numbers are 78.75, 157.5, and 315, respectively. The magnitude of the velocity has been normalized to a maximum of 1.0. From the figures, it is observed that there is a vortex formation or recirculation zone in the area where the magnetic field is located. It is shown that the vortex increases in size in both height and length, starting with lower to higher magnetic field intensity. It is interesting to notice that the vortex’s general shape follows that of an airfoil. Although not shown in Figure 2, it should be remarked that without the magnetic field ($\bar{B_{o}}$ = 0), the velocity of the flow is the same as that of the fully developed flow at the entrance.

**Figure 3:**
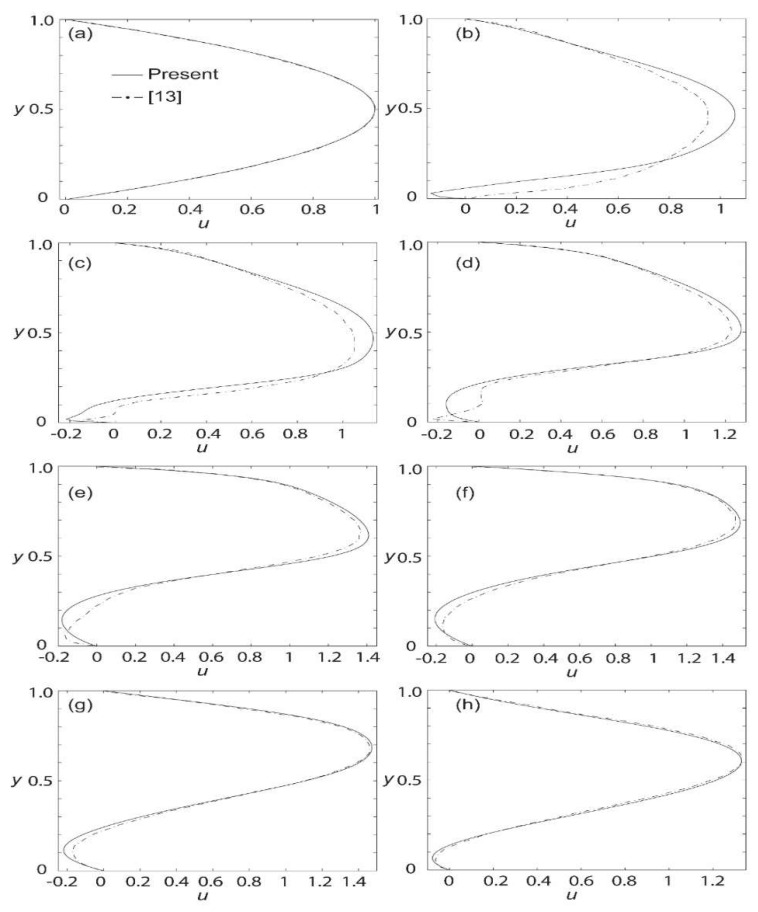
Verification of axial velocity profiles at various parts of the channel for $\bar{B_{o}}\;$ = 8T and *Re* = 250 against [13]: *x* = (**a**) 0; (**b**) 2.5; (**c**) 2.6; (**d**) 2.8; (**e**) 3.1; (**f**) 3.5; (**g**) 4.5; and (**h**) 5.5.

Figure 3a–h show the currently computed axial velocity profiles compared to Loukopoulos and Tzirtzilakis [15] at different locations along the channel for $\bar{B_{o}\;}$= 8 T. At *x* = 0, the axial velocity is identical due to the boundary condition imposed. At *x* = 2.5, a slight deviation can be visualized with the sharp edge in the velocity profile at the bottom of the channel. Moving further to *x* = 2.6, the result seems to coincide nicely, but at *x* = 2.8, the result seems to deviate again slightly. It is suspected that this small discrepancy is due to the difference in the recirculation region approximated by [15] and the current study. Correspondingly, this would then offset the location of the velocity gradient. This is especially true near the magnetic source. It is noted that far from the magnetic source, the agreement is good. For the flow pattern from *x* = 3.1 until *x* = 5.5, the results match rather closely. It is also noted that the outlet’s velocity reverts to that of a fully developed flow. 

In general, it can be seen that starting at *x* = 2.6, the maximum axial velocity increases from a dimensionless value of 1 to about 1.1 and continues to increase until it peaks at 1.45 at *x* = 5.5. In the recirculation zone where backflow occurs, it can be observed that the axial velocity is minimal at approximately −0.2. Overall, the computed profiles generally agree with those presented by Loukopoulos and Tzirtzilakis [13], verifying the currently formulated model. In the remainder of this paper, the profile and contour plots for axial velocity, temperature, and skin friction coefficient are further explored in the cases of different magnetic intensities.

### 3.2. Velocity

**Figure 4:**
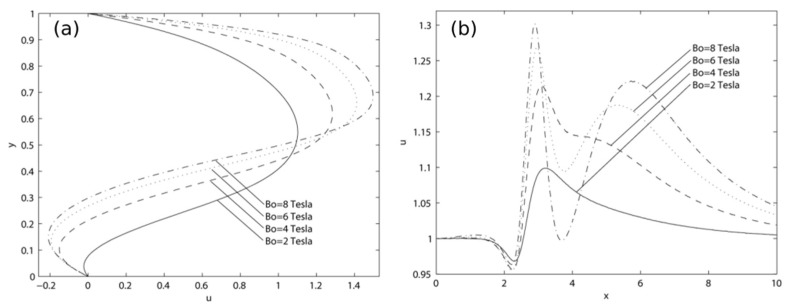
Axial velocity profiles at (**a**) *x* = 3.5; and (**b**) mid-span of the channel when subjected to various magnetic field strengths with *Re* = 250.

Figure 4a shows the axial velocity at *x* = 3.5 for various magnetic field strengths. For $\bar{B_{o}}\;$= 2T, the maximum axial velocity is about 1.1. It can be observed that the axial velocity increases to 1.25, 1.4, and 1.45 for $\bar{B_{o}}$ of 4 T, 6 T, and 8 T, respectively. Furthermore, with the increase in the magnetic field strength, backflow starts to form at the lower part of the channel. The minimum value reaches approximately −0.2 at the magnetic field strength of 8 T. Therefore, it is noted that by increasing the magnetic field strength, the velocity at the upper part of the channel increases, but the velocity is reduced at the lower part, where the magnetic field is imposed. Moreover, the axial velocity distribution along the middle span of the channel is shown in Figure 4b for various magnetic field strengths. The channel has a unit velocity at the inlet, but it suddenly drops at *x* = 2.5 followed by a steep rise in magnitude. The velocities peak at 1.1, 1.21, 1.26, and 1.3 for $\bar{B_{o}}$ of 2 T, 4 T, 6 T, and 8 T, respectively. Upon reaching the maximum value at the location between *x* = 3 and *x* = 4, the axial velocity plummets down with slight oscillations before exiting the channel. It can also be witnessed that the outlet velocity differs for each magnetic field strength. This is due to the inadequate length of the channel to allow for the complete development of the flow.

### 3.3. Temperature Profile

**Figure 5:**
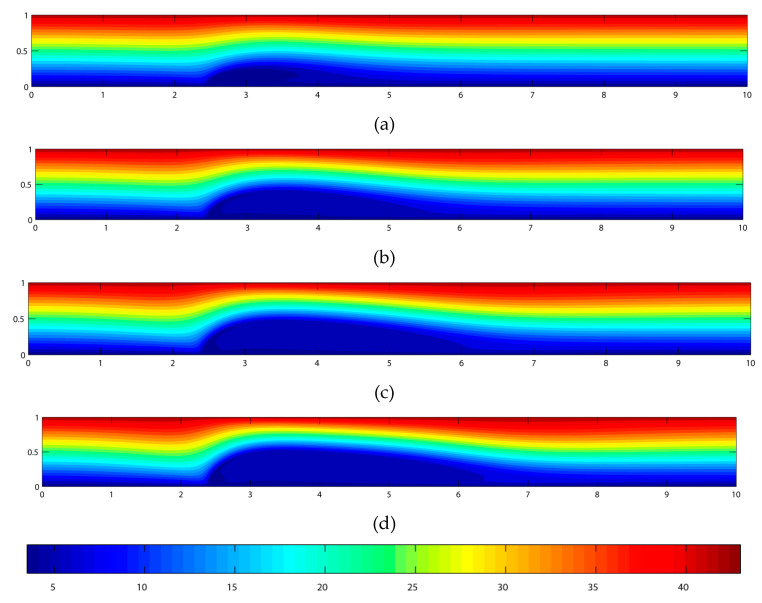
Temperature contour plots for biomagnetic flow subjected to $\bar{B_{o}}$ of (**a**) 2 T; (**b**) 4 T; (**c**) 6 T; and (**d**) 8 T.

The dimensionless temperature contours for various magnetic field strengths are shown in Figure 5. From the contour plot, it can be seen that there is a disturbance in the temperature field after the point of the magnetic source, and the disturbance becomes greater with the increase in the magnetic field strength. The disturbance of the temperature field follows the same pattern as the axial velocity. With an increase in the magnetic field strength, the height and length of the disturbance are extended accordingly.

**Figure 6:**
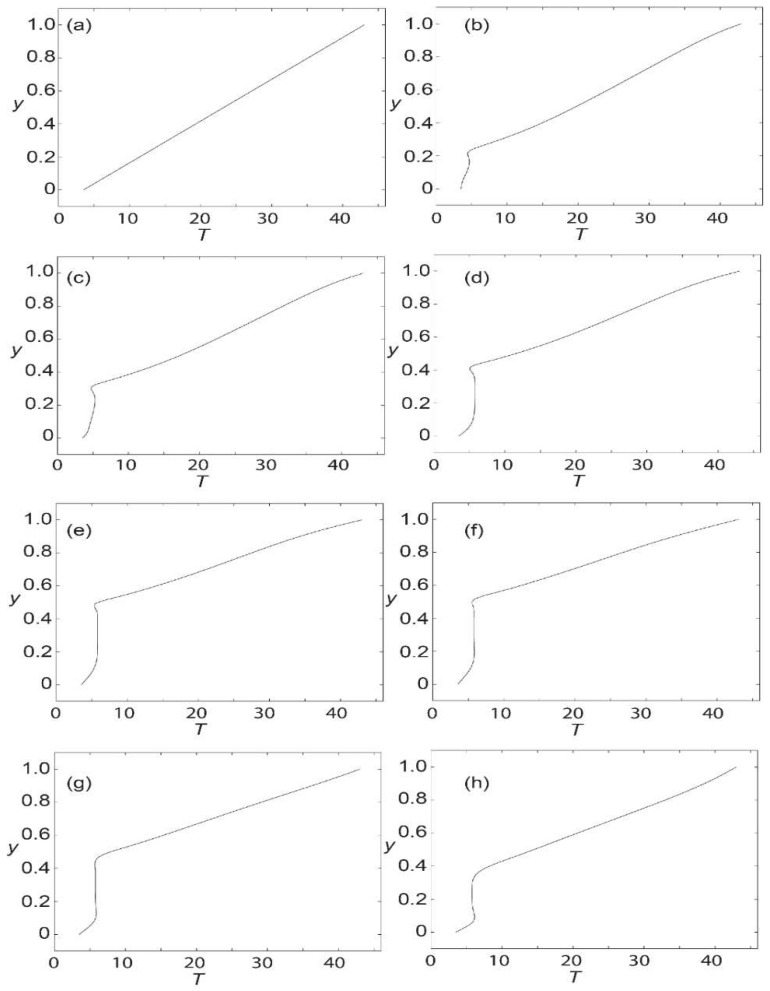
Temperature profiles at various parts of the channel subjected to $\bar{B_{o}\;}$ = 8 T and *Re* = 250 at *x* = (**a**) 0; (**b**) 2.5; (**c**) 2.6; (**d**) 2.8; (**e**) 3.1; (**f**) 3.5; (**g**) 4.5; and (**h**) 5.5.

To further investigate this, the temperature profiles at various parts of the channel for $\bar{B_{o}}\;$= 8T and *Re* = 250 are presented in Figure 6. The temperature is linearly distributed from the lower wall to the upper wall at the inlet. A sudden jolt in the temperature profile is evidenced at *x* = 2.5. Here, the temperature remains constant at about 5 °C from *y* = 0 to *y* = 0.2. The same trend continues for the temperature profile at locations *x* = 2.6, 2.8, 3.1, and 3.5, where the constant temperature continues to retain its value from *y* = 0 until *y* = 0.5. The constant temperature value decreases from *x* = 4.5 and beyond. This flow trend indicates that with the introduction of a magnetic source, there is a disturbance in the temperature field at the location where the magnetic source is imposed. The temperature disturbance extends with the increase of the magnetic field strength. It is also observed that the temperature disturbance, due to the magnetic field source, results in a slight deviation in the temperature profile when compared to those at the entrance and the exit. In the absence of a magnetic field, the contours of the temperature are linearly distributed from the lower to the upper walls.

**Figure 7:**
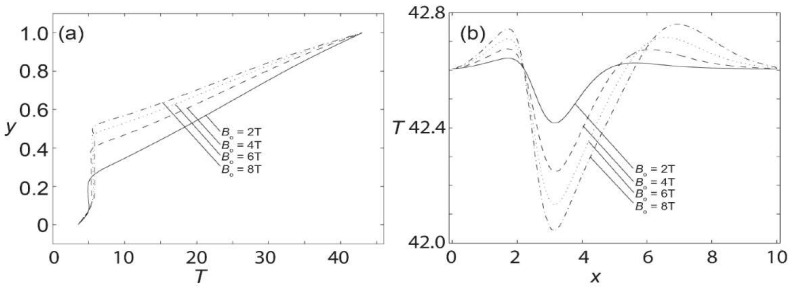
Temperature profiles at (**a**) *x* = 3.5; and (**b**) mid-span of the channel when subjected to various magnetic field intensities with *Re* = 250.

Next, Figure 7a shows the temperature profiles for various magnetic fields at *x* = 3.5. It is evidenced from the figure that the increase in magnetic field strength affects the temperature field. The effect is more obvious for the magnetic field strength of 8 T, affecting the area from *y* = 0.1 to *y* = 0.5, compared to the magnetic strength of 2 T, where it only affects the temperature from *y* = 0.1 to *y* = 0.25. Moreover, the temperature distribution for various magnetic strengths along the middle of the channel, is shown in Figure 7b. It is also shown that the shape of the temperature distribution along the middle of the channel remains qualitatively the same for all the magnetic field strengths. The disturbance starts at the inlet of the channel until *x* = 2. Then, the value drops drastically to its minimum at *x* = 3, before increasing steadily to its maximum between *x* = 4 and *x* = 8 for all magnetic field strengths.

### 3.4. Skin Friction Coefficient

**Figure 8:**
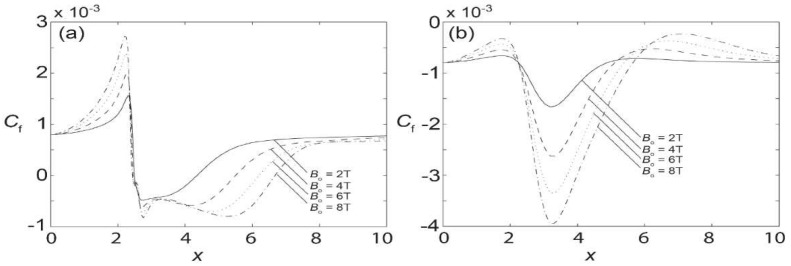
Skin friction coefficient along the channel when subjected to various magnetic field intensities at *Re* = 250: (**a**) lower wall; and (**b**) upper wall.

One of the most important fluid characteristics that is customarily examined is the skin friction coefficient. The skin friction coefficient, *C_f_*, is given by the formula:

(28) $$C_{f}=\frac{2\bar{\tau }}{\bar{\rho }{\bar{u_{r}}}^{2}}$$

where $\bar{\tau }={\bar{\mu }\left(\partial \bar{u}/\partial \bar{y}\right)|}_{\bar{y}=\bar{0},\bar{h}}$ is the wall shear stress. Figure 8 shows the local skin friction coefficient for the upper and lower walls in the presence of $\bar{B_{o}}$, of 2 T, 4 T, 6 T, and 8 T. A comparison between the upper and lower walls suggests that the skin friction coefficient has more influence on the lower wall in the range of *x* = 2 to *x* = 4, where the magnetic source is placed. It is also noteworthy that while the lower wall has the variation of skin friction in the positive and negative range, the upper wall only exhibits negative skin friction coefficients. Furthermore, with the increase in magnetic strength, the maximum and minimum values are enhanced and extended, but the shapes of the skin friction coefficients remain qualitatively the same. The maximum skin friction coefficient occurs at *x* = 2.1, while the minimum can be found at *x* = 3.1 for $\bar{B_{o}}\;$= 8 T. These locations are generally followed in the cases of other magnetic intensities as well.

For the biomagnetic fluid flow in a channel under the influence of spatially varying magnetic fields, it is evidenced that the formulation derived from the mixed finite element method is verified and shows vitality for modeling the biomagnetic fluid characteristics. It is observed that with the introduction of the magnetic field at the lower part of the channel, the flow is appreciably disturbed. The velocity at the lower wall, where the magnetic source is located, is reduced whilst the velocity at the upper parts increases, resulting in the formation of a vortex. Furthermore, the vortex size increases with the magnetic field strength. Apart from the velocity, it is also noticed that the amplification of the magnetic field strength increases disturbances in the temperature, especially at the location of the magnetic source. Lastly, the skin friction coefficient is greater at the lower wall near the magnetic source, than at the upper wall.

## 4. Conclusions

The mixed formulation of the finite element model for the Navier–Stokes equations, containing energy and magnetic effects, was successfully developed. The formulation consisted of coupling between the Navier–Stokes equation, heat transfer, ferrohydrodynamics, and magnetohydrodynamics, the last two components of which are the subsets of the electromagnetic discipline. Additionally, the Galerkin method was employed to discretize the governing equations, resulting in a nonlinear system of simultaneous equations. The modeling of the biomagnetic fluid flow in a straight rectangular channel, subjected to spatially varying magnetic fields, was then conducted. Due to a sharp gradient in the magnetic field, the effect of Lorentz force on the flow was small, hence neglected, for the rest of the study.

− Excellent agreement was exhibited when comparing the computed result from the current model against that from the literature.
− It was found that when subjected to a spatially varying magnetic field, a vortex arises upstream from the magnetic source.
− The size of the vortex increases as the magnetic field strength increases.
− Furthermore, the temperature around the magnetic source was observed to be considerably disturbed.
− Skin friction increased at the upper and lower walls due to the existence of varying magnetic field intensities.
− It is noted that the Newtonian assumption of the blood in this study is only valid for blood in large arteries. For blood in narrow arteries, the behavior of the blood is closer to that of a non-Newtonian fluid. Thus, non-Newtonian fluid is one potential subject for further development of the model. A high-gradient magnetic field renders Lorentz force insignificant to the flow.
− It is evidenced from the literature that the Lorentz force could play an imperative role in a constant magnetic field. Thus, for future study, the inclusion of the Lorentz force should be considered by applying several types of magnetic field gradients, so that the effects of Lorentz force are more apparent.
− Two-dimensional cases such as those used in the present study offer cheaper computational time and storage costs, but a realistic case usually involves a full three-dimensional geometry. Therefore, the three-dimensional geometry of the BFD problems is one prospective area for study in the future.

## Figures and Tables

**Table 1:** Dimensional variables for biomagnetic flow in a rectangular channel.

Parameter	Symbol	Unit	Value
Density	$\bar{\rho }$	kg/m^3^	1050
Viscosity	$\bar{\mu }$	kg/(ms)	3.2 × 10^−3^
Thermal conductivity	$\bar{k}$	J/(msK)	2.2 × 10^−3^
Heat capacity	$\bar{C_{p}}$	J/(kgK)	14.65
Reference velocity	$\bar{U_{r}}$	m/s^−1^	3.81 × 10^−2^
Magnetic permeability of vacuum	$\bar{\mu_{o}}$	N/A^2^	4π × 10^−7^
Temperature of upper wall	$\bar{T_{u}}$	K	316.15 (43 °C)
Temperature of lower wall	$\bar{T_{l}}$	K	276.65 (3.5 °C)
Channel height	$\bar{H}$	m	2.0 × 10^−2^
Channel length	$\bar{L}$	m	2.0 × 10^−1^
Prandtl number	$P_{r}$		20
Temperature number	ε		8
Eckert number	$E_{c}$		2.476 × 10^−6^
Reynold number	Re		250
Magnetic number (FHD)	$Mn_{F}$		314

## Data Availability

Not applicable.
